# Female Community Health Volunteer-led intervention for hypertension prevention and control in rural Nepal: A hybrid type 2 effectiveness-implementation design

**DOI:** 10.1371/journal.pgph.0006057

**Published:** 2026-07-06

**Authors:** Bihungum Bista, Sushmita Mali, Kjersti Mørkrid, Meghnath Dhimal, Biraj Man Karmacharya, Archana Shrestha

**Affiliations:** 1 Kathmandu University School of Medical Sciences (KUSMS), Dhulikhel, Nepal; 2 Institute for Implementation Science and Health (IISH), Kathmandu, Nepal; 3 Division for Health Services, Norwegian Institute of Public Health (NIPH), Oslo, Norway; 4 Department of Global Public Health and Primary Care, University of Bergen, Bergen, Norway; 5 Nepal Health Research Council (NHRC), Kathmandu, Nepal; 6 Dhulikhel Hospital, Dhulikhel, Nepal; University of Leicester, UNITED KINGDOM OF GREAT BRITAIN AND NORTHERN IRELAND

## Abstract

Hypertension is a leading cause of cardiovascular disease and premature mortality, disproportionately affecting low- and middle-income countries such as Nepal, where awareness, treatment, and control remain low. Female Community Health Volunteers (FCHVs) have been effective in maternal and child health programs and may support noncommunicable disease management. This hybrid type 2 effectiveness-implementation study evaluated the effectiveness and implementation outcomes of an FCHV-led intervention for hypertension prevention and control in rural Nepal. We conducted a cluster randomized controlled trial (ClinicalTrials.gov: NCT06163859) in Namobuddha Municipality, Nepal, randomizing 12 public primary healthcare facilities (1:1) to intervention or control arms. In intervention clusters, 34 FCHVs delivered a community-based hypertension program through bi-monthly group sessions over three months, including blood pressure monitoring, health education, lifestyle counseling, medication adherence support, and referral for uncontrolled hypertension. Implementation strategies were developed using an Implementation Research Logic Model and stakeholder consultations. Adults aged 30–75 years with elevated blood pressure were recruited through community screening. Implementation outcomes were assessed using the RE-AIM framework and effectiveness outcomes at three months. Analyses followed intention-to-treat principles using generalized estimating equations. Reach was 56.9%, with higher male participation than female participation (63.5% vs. 50.4%; p = 0.045). Adoption was 100% across facilities. Fidelity was moderate, with 76.5% attending at least four of six sessions and 71.1% of meetings achieving at least 70% attendance. No significant differences were observed in systolic blood pressure, diastolic blood pressure, or hypertension control. However, the intervention significantly improved hypertension knowledge and treatment adherence. The FCHV-led intervention was feasible and achieved moderate reach, universal adoption, and reasonable fidelity. Although blood pressure outcomes did not improve over three months, longer interventions with sustained support may improve clinical outcomes and support scale-up within Nepal’s noncommunicable disease program.

## Introduction

Hypertension is a major global public health challenge and a leading cause of cardiovascular disease, stroke, kidney failure, and premature mortality, affecting an estimated 1.3 billion adults worldwide and contributing to about 10.8 million deaths annually [1]. Timely screening, initiation of treatment, and sustained blood pressure control are critical to reducing hypertension-related morbidity and mortality [2,3].

The burden of hypertension is raising rapidly in low- and middle-income countries (LMICs), where health systems often face significant resource constraints, the burden of hypertension is growing rapidly [4] and Nepal is no exception. The World Health Organization (WHO) Nocommunicable diseases(NCDs) STEPS Survey 2019 found that approximately 25% of adults aged 15–69 years in Nepal were hypertensive, with only 56% of them ever having had their blood pressure measured before the survey and just 20% of those diagnosed had adequate blood pressure control [5,6].The Government of Nepal has introduced the WHO’s Package of Essential Noncommunicable Disease Interventions (PEN) at the primary care level [7] to extend the reach of hypertension management. However, its implementation remains limited due to systemic barriers such as poor access to health services, limited awareness at the community level, and suboptimal adherence to prescribed medication and lifestyle modification [8–10] The mobilization of community health workers (CHWs) has emerged as one of the cost-effective, sustainable, and locally adaptable approaches—especially in LMICs to address hypertension screening and management challenges. CHW-led blood pressure monitoring and counseling support for medication adherence and healthy behaviors, in addition to enhancing the linkage to care, have demonstrated success [11–13]. In rural communities in South Asia (Bangladesh, Pakistan, and Sri Lanka), a cluster-randomized trial showed that a multicomponent intervention led by trained government community health workers was effective in reducing systolic blood pressure, with a mean reduction 5.2 mm Hg greater in the intervention group than in the usual care group [14].

In Nepal, Female community health volunteers (FCHVs) are a nationally recognized cadre of Community health workers (CHW) who have contributed significantly to maternal, newborn, and child health (MNCH) programs for over three decades [15–17]. Their deep roots in the community, long-standing trust, and volunteer spirit make FCHVs well-suited to expand into NCD prevention and control. The COBIN trial demonstrated FCHV-led interventions’ effectiveness in hypertension management, achieving a 4.9 mm Hg greater mean systolic blood pressure reduction in hypertensive participants after 12 months [18]. However, FCHVs have not been actively engaged in NCD-related services, including delivery of WHO PEN [19].

So, in this context, we implemented a community-based FCHV-led hypertension prevention and control in a rural municipality of Nepal. The core intervention consisted of FCHV-facilitated group sessions that included blood pressure monitoring, structured health education, counseling on risk factors and lifestyle modification, medication adherence support, and referral of participants with uncontrolled blood pressure to health facilities.

To ensure successful delivery and sustainability, we implemented theory-informed strategies designed to address key barriers within the health system and FCHV levels. These included competency-based FCHV training and supportive supervision to address gaps in knowledge, confidence, and workload-related challenges, with the rationale that improved capacity would enhance fidelity and acceptability [20–23]. We provided digital blood pressure devices and literacy-appropriate flip chart to reduce implementation complexity, standardize counseling practices, and improve efficiency and community trust, thereby strengthening implementation fidelity [22]. Coalition building through FCHV-led hypertension groups leveraged strong community trust and alignment with provincial and WHO-supported programs to promote peer support, sustained engagement, and maintenance of behavior change [22,24–26]. Finally, network weaving through regular FCHV–facility coordination meetings aimed to address fragmented referral pathways and poor continuity of care, with the rationale that strengthened community–facility linkages would enhance implementation, effectiveness, and long-term sustainability [27].

While prior study in Nepal [18], has shown that FCHV-led approach can improve hypertension outcomes, the implementation processes and outcomes—such as fidelity, reach, adoption, and sustainability—remain poorly documented. Understanding how and why such interventions work in real-world primary healthcare settings is essential to inform scale-up and integration within the national NCD program. So, this study aims to evaluate the mobilization of FCHVs for hypertension prevention and control in a rural municipality of Nepal.

## Methods

### Ethics statement

The Ethical Review Board of the Nepal Health Research Council approved the study (Reference No. 459/2023). Further, we obtained written informed consent from all participants. For participants who were illiterate, a trained study team member read the consent form aloud, and participants provided consent using a fingerprint in the presence of a witness.

### Study site and settings

This study used a hybrid type 2 cluster randomized controlled trial in Namobuddha Municipality, Kavre District, Bagmati Province, Nepal. The municipality has a population of approximately 27,000, served by 12 health facilities and 82 FCHVs [28,29]. This site was selected in consultation with local and provincial health authorities for its readiness to implement community-based interventions.

The trial is registered at ClinicalTrials.gov (NCT06163859) [30]. This study followed the Standards for Reporting Implementation Studies (STaRI) guideline and CONSORT [31,32].

### Design

This study employed a parallel cluster-randomized controlled trial. We randomized twelve public health facilities (1:1) to the intervention (n = 6) or control (n = 6) arm using a random sequence generated in R. A statistician developed ten randomization sets. A health coordinator not involved in the research publicly conducted the allocation using sealed envelopes and selected one randomization set. Blinding was not feasible after allocation.

### Study population and recruitment

The study population included two key groups: FCHVs and individuals with high blood pressure. We included FCHVs who were pre-assigned to their respective health facilities based on existing government-defined catchment areas of Namobuddha Municipality. We enrolled the 34 FCHVs working under the 6 different health facilities of the intervention arm, and who were willing to participate in this study.

For high blood pressure individuals, inclusion criteria were: age between 30 and 75 years (as hypertension typically develops after age 30, and individuals above 75 years may face mobility limitations and require different counseling approaches), residence in Namobuddha Municipality for at least six months, an identified as high blood pressure (SBP ≥ 130 mmHg or DBP ≥ 80 mmHg) or current use of antihypertensive medication [33]. We specifically enrolled individuals meeting the blood pressure threshold (SBP ≥ 130 mmHg or DBP ≥ 80 mmHg) since the study aimed to both prevent the progression of hypertension and support its management through counseling. We excluded pregnant and postpartum women (≤8 weeks) due to physiological changes affecting blood pressure, individuals unable to complete data collection to ensure validity, and those with mental incapacitation who were uncapable to provide informed consent firmly.

We identified individuals with hypertension who met the inclusion criteria by conducting 20 community-based hypertension screening camps in collaboration with health facility staff and Female Community Health Volunteers (FCHVs) across the municipality. We invited eligible individuals to participate and recruited a total of 520 participants from both intervention and control sites after obtaining written informed consent. The study was conducted from January 2023 to May 2025. Participant recruitment took place between September and October 2024, and follow-up was conducted in February and March 2025. For illiterate participants, a trained member of the study team read the consent form aloud and obtained informed consent using a fingerprint in the presence of an impartial witness. We did not provide any financial incentives to participants.

### Intervention and implementation strategies

#### Intervention.

We implemented a community-based hypertension intervention in Namobuddha Municipality by mobilizing 34 FCHVs as frontline implementers. Each FCHV formed a group of 7–16 participants and conducted one-hour, bi-monthly sessions over three months. The sessions included blood pressure monitoring, structured health education on hypertension and its risk factors, lifestyle modification counseling, medication adherence support, and referrals to primary care for participants with uncontrolled blood pressure.

FCHVs were chosen as intervention agents due to their trusted role and strong presence within the community, which facilitates engagement and behavior change in case of Nepal [15–17], globally also role of CHW against hypertension is well established [11–13,34]. Regular blood pressure monitoring increases awareness and promotes timely management [35], while structured education grounded in Social Cognitive Theory enhances knowledge, builds self-efficacy, and encourages healthy behaviors through peer learning and reinforcement [24,25]. Medication counseling supports adherence, and referrals ensure continuity of clinical care [36–40]. The CHW group-based delivery model fosters observational learning and social support, offering a low-cost, multifaceted approach that addresses both behavioral and clinical dimensions of hypertension management [41,42].

#### Implementation strategies.

We developed an Implementation Research Logic Model (IRLM)(S1 Fig) [43], and identified the key determinants, implementation strategies, mechanisms, and outcomes. For this, we conducted two rounds of consultative meeting with health workers, one round of cognitive interview meeting with FCHVs from different municipality, and two round of meeting with health coordinator. Furthermore, we also had consultative meeting with responsible authority of government of officials from federal, provincial and local level. Together, they provided the feedback and other valuable other locally adapted materials, that assisted us to develop locally adapted effective proven health education media, i.e., flip charts materials [44] and delivery approaches to line with FCHVs’ literacy levels, workload, and local sociocultural context.

We explicitly designed our implementation strategies to address key barriers and leverage facilitators. The strategies targeted both inner and outer setting barriers identified during formative assessments and prior evidence on FCHV-led interventions in Nepal.

First, to strengthen FCHVs’ technical capacity, we conducted a one-time, three-day competency-based training before implementation. The training enhanced FCHVs’ skills in blood pressure measurement, behavior change communication, documentation, and goal setting.

Second, we provided clinical and technical infrastructure support by equipping each FCHV with a digital automated blood pressure monitor and literacy-appropriate intervention materials.

Third, we built coalitions by establishing and facilitating FCHV-led hypertension groups (FCHV-HTN groups) consisting of individuals with or at risk of hypertension. These groups met twice monthly throughout the intervention period to promote behavior change, medication adherence, and self-care practices.

Fourth, to strengthen linkages between community and facility levels, we implemented a network weaving strategy through monthly coordination and referral meetings between FCHVs and health facility in-charges, aiming to improve referral systems, enhance communication, and create feedback loops for shared problem-solving.

### Control Group

Participants in the control group received the routine care through Nepal’s public health system, primarily the WHO PEN package is a government-adopted strategy to manage common Non-Communicable Diseases (NCDs) including hypertension at primary health centers, providing evidence-based protocols, training for health workers, and guidelines for essential medicines [7]. In addition, the Bagmati Kavrepalanchok Hypertension Care Cascade Initiative (BKHCCI) supported hypertension care in the Namobuddha by training health workers, authorizing paramedics to initiate and management hypertension, and providing digital blood pressure monitors [26,27].

### Sample Size

We determined the sample size assuming a standard deviation of 14 mmHg for systolic blood pressure, targeting 80% statistical power and a two-sided 5% significance level to detect a minimum mean difference of 5 mmHg, between the intervention and control arms resulting sample size of 400. Then, we adjusted for clustering effects using a design effect of 1.61 that assumed an average cluster size of 43, an intraclass correlation coefficient of 0.0146), and accounted for a 20% loss to follow-up [45,46] that resulted the sample size of 520 for 12 health facilities.

### Outcomes

This study assessed the outcomes at two levels: implementation and clinical. Implementation outcomes were assessed using RE-AIM framework [47]. Within the RE-AIM framework, information related to maintenance was not included in this study, as this dimension is typically assessed over a longer period. The current study duration was not sufficient to adequately measure maintenance outcomes.

### Implementation outcomes

#### Reach.

Reach was defined as the proportion and characteristics of eligible individuals who received the intervention, operationalized as the percentage of eligible participants who attended at least one FCHV session. To assess reach, we examined both participation rates and the representativeness of participants. Data sources included FCHV session logs to quantify reach, while the baseline and endline surveys provided socio-demographic profile of participants, who participated in the FCHV sessions.

#### Effectiveness.

The primary effectiveness outcome was the change in mean systolic blood pressure at 3 months. Trained research assistants measured blood pressure using a digital Omron HEM 8712 device. We recorded blood pressure three times at one-minute intervals after five minutes of rest in a seated position as per the guideline and used the average of the last two readings for analysis [48].

Secondary outcome included diastolic blood pressure and hypertension control defining control status SBP < 140 mm Hg and DBP < 90 mm Hg at three months [49]. Further, we also hypertension knowledge and medication adherence using valid and reliable measurement tools adapted to the Nepalese context. The Hypertension Knowledge-Level Scale (HK-LS) score, includes six sub-dimensions and scores ranging from 0 to 22; where higher scores indicate greater knowledge [50]. We assessed Medication adherence with the medication subscale of the Hill-Bone Compliance to High Blood Pressure Therapy Scale; lower scores indicate better adherence. We assessed overall compliance to hypertension management using the full Hill-Bone scale, which covers appointment keeping, dietary practices, and medication adherence; scores range from 14 to 56, with lower scores reflecting higher compliance [51–53]. Similarly, we also assessed dietary behavior using the (range 0–18), where higher scores indicate better adherence to healthy dietary practices [54]. We also measured body mass index (BMI) calculated from standardized height and weight measurements using Seca equipment following standard measurement guidelines [48], and categorized participants as normal weight (18.5 - 25 kg/m²) or overweight/obese (≥25 kg/m²).

#### Adoption.

Adoption was defined as the proportion of eligible health facilities that agreed to participate in the study. Participation was confirmed through signed minutes from meetings between the municipal health coordinator and facility representatives, indicating formal approval to implement the program.

#### Implementation.

We assessed implementation fidelity by measuring the proportion of participants who attended four or more health education sessions, the percentage of group meetings with at least 70% of enrolled participants present, and the proportion of monthly meetings held by health facilities with FCHVs. FCHVs and health workers recorded these details in their session logs and reporting form respectively, which we used to evaluate both participant engagement and adherence to the planned group-based delivery approach.

### Data collection tools and techniques

Well-trained interviewers conducted face-to-face interviews using modified standardized questionnaires adapted from the WHO STEPS questionnaire (version 3.2) [48], administered in Nepali, with responses entered into a REDCap form. The questionnaire included:

(a) Socio-demographic Information: Gender (male/female), age (in years), ethnicity (Brahmin/Chhetri, Janajati, Dalit, others), marital status (unmarried, married, ever married), educational status (illiterate/less than primary, primary, secondary, bachelor and higher), and religion (Hindu, Buddhist, others).
(b) Lifestyle Information: Tobacco use (current smokers, past smokers, non-smokers) and alcohol consumption (abstainers, non-drinkers in past 12 months, current drinkers) were categorized per WHO STEPS methodology [48]. Physical inactivity was assessed using the Global Physical Activity Questionnaire (GPAQ), classifying individuals as inactive if below 600 MET minutes per week [55].

### Statistical analysis

This study was evaluated implementation outcomes using the RE-AIM framework (Reach, Effectiveness, Adoption, Implementation, and Maintenance). We summarized implementation data descriptively, reporting continuous variables as means, standard deviation and categorical variables as frequencies (%).

Primary outcomes comprised change in systolic blood pressure (SBP). Secondary outcomes included, change in diastolic blood pressure (DBP), and hypertension control (SBP < 140 mm Hg and DBP < 90 mm Hg), BMI category (overweight/obese vs. normal/underweight), hypertension knowledge, diet, and medication adherence scores.

We conducted an intention-to-treat (ITT) analysis using generalized estimating equations (GEE), adjusting for baseline outcome values and clustering at the health facility level [56,57], without imputing missing data. We also conducted a secondary as-treated analysis (S1 Table) to examine dose–response effects, categorizing intervention exposure as “none,” “low,” or “high” based on session attendance. We applied linear GEE models (Gaussian family with identity link) for continuous outcomes to estimate adjusted mean differences and modified Poisson GEE models (log link with robust variance) for binary outcomes to estimate relative risks [58]. We assumed an exchangeable correlation structure and used Huber–White robust standard errors to account for potential misspecification of the correlation structure.

We also performed sensitivity analyses by excluding outliers (beyond 1.5 × interquartile range). We used two-sided tests with P < 0.05 denoting significance. We conducted all the analyses in R (version 4.1.1) using packages gee (modeling), haven (data import), dplyr (management), flextable, and officer (tables).

## Results

**Fig 1:**
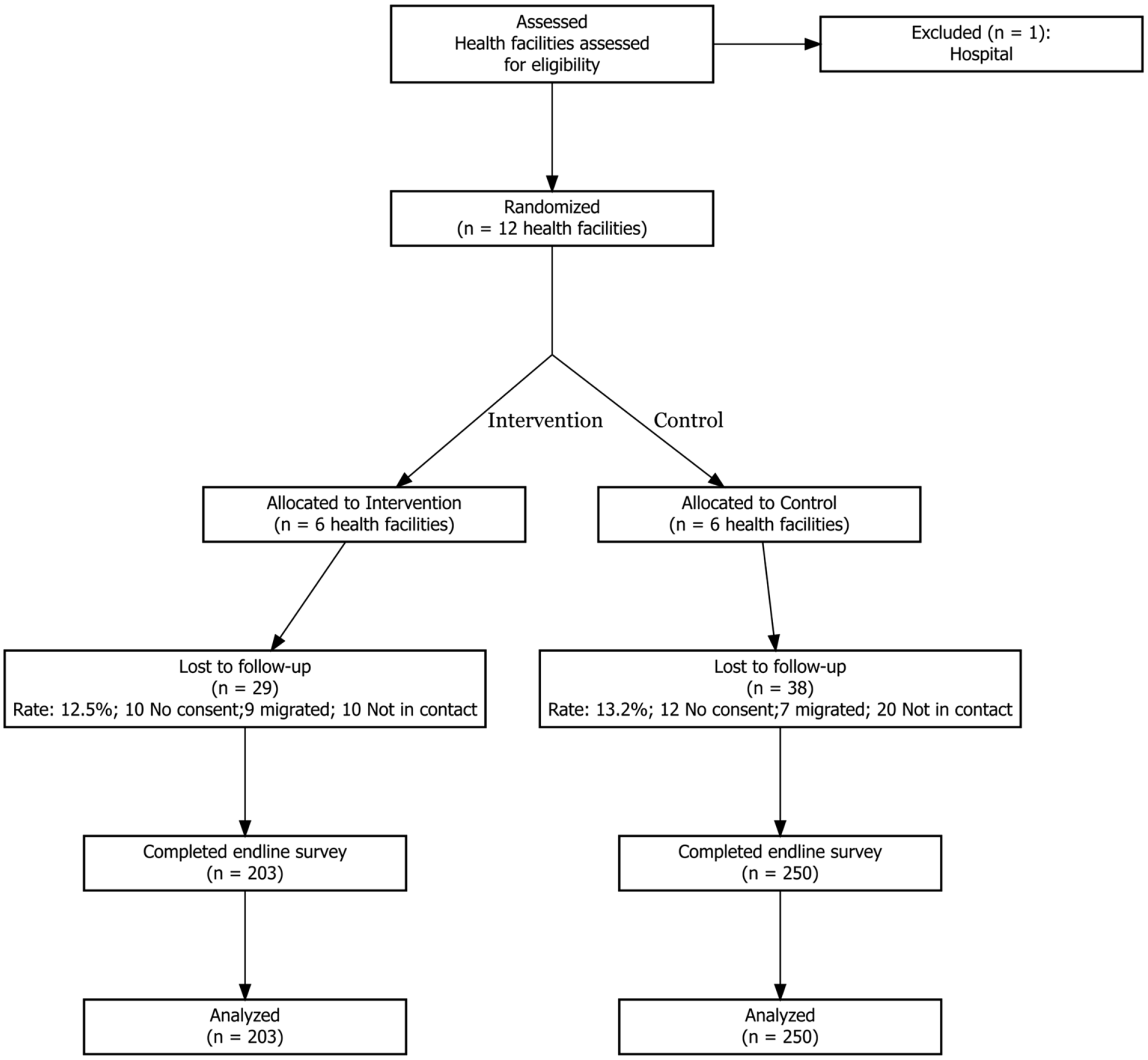
Flow diagram of study process.

We enrolled 520 participants according to their health facility’s random allocation, resulting in 232 participants in the intervention arm and 288 in the control arm. (Fig 1) During follow-up, 12.5% (n = 29) of participants in the intervention arm were lost to follow-up, primarily due to withdrawal of consent (n = 10), migration (n = 9), and loss of contact (n = 10). In the control arm, 13.2% (n = 38) of participants were lost, due to withdrawal of consent (n = 12), migration (n = 7), and loss of contact (n = 20). We completed endline surveys for 203 participants in the intervention arm and 250 in the control arm.

Participant characteristics were balanced between the intervention and control groups at baseline (Table 1). Mean age was 59.7 ± 10.5 years in the intervention group and 61.2 ± 10.3 years in the control group. Half of the participants were women (50.4% in intervention arm vs. 50.0%in control arm). The largest ethnic group was Janajati (49.6% in intervention arm vs. 56.9% in control arm), followed by Brahmin/Chhetri (43.9%in intervention vs. 38.9% in control); most participants were Hindu (66.8%in intervention arm vs. 68.1% in control arm). Approximately half were illiterate (50.7% in intervention arm vs. 49.7% in control arm), and the majority were self-employed or engaged in business (78.3% in intervention arm vs. 76.0% in control arm). Behavioral risk factors (current tobacco use, former tobacco use, and recent alcohol consumption) were also comparable between the groups.

**Table 1:** Baseline characteristics of participants.

Variable (Category)	Intervention(n(%)) N = 232	Control(n(%)) N = 288	Total(n(%)) N = 520	P-value
**Age** (mean,sd)	59.67 ± 10.5	61.19 ± 10.3	60.51 ± 10.4	0.09
**Gender**				
Male	115 (49.5%)	144 (50.00%)	259 (49.8%)	0.56
Female	117 (50.4%)	143 (50.00%)	261 (50.2%)	
**Ethnicity**				
Brahmin/Chettri	102 (43.9%)	112 (38.9%)	214 (41.2%)	0.18
Janjati	115 (49.6%)	163 (56.9%)	278 (53.7%)	
Dalit	15 (6.5%)	12 (4.2%)	27 (5.2%)	
**Religion**				
Hindu	155 (66.8%)	196 (68.1%)	351 (67.5%)	0.79
Buddhist	73 (31.4%)	85 (29.5%)	158 (30.4%)	
Christian	4 (1.7%)	7 (2.4%)	11 (2.1%)	
Education Level*				
Illiterate	117 (50.4%)	143 (49.7%)	260 (50.1%)	0.81
Primary	69 (29.7%)	81 (28.1%)	148 (28.7%)	
Secondary or higher	46 (19.8%)	64 (22.2%)	110 (21.2%)	
**Occupation**				
Employed (Formal Sector)	3 (1.3%)	11 (3.8%)	14 (2.7%)	0.19
Self-Employed/Business	184 (78.3%)	219 (76.0%)	403 (77.1%)	
Non-Economically Active	45 (19.4%)	58 (20.1%)	103 (19.8%)	
Tobacco User*				0.08
Never tobacco user	137 (59.3%)	143 (49.7%)	280 (53.9%)	
Former tobacco user	45(19.5%)	75(26.0%)	120(23.1%)	
Current tobacco user	49 (21.2%)	85 (29.5%)	145 (27.0%)	
**Alcohol Use Status (Past 1 Month)**				
Abstainer	167 (80.7%)	189 (76.5%)	356 (78.4%)	0.40
Non-user (past 1 month)	10 (4.8%)	15 (6.1%)	24 (5.5%)	
User (past 1 month)	30 (14.5%)	43 (17.4%)	73 (16.1%)	

* 1 case missing.

### Implementation outcomes

#### Reach.

Of the 232 eligible adults with hypertension invited to participate, 132 (56.9%) attended at least one FCHV session.

Non-attendees and attendees of health education sessions showed similar baseline characteristics (Table 2). Mean age was 60.8 ± 10.2 years among non-attendees and 59.6 ± 10.9 years among attendees (p = 0.25). A significantly higher proportion of males attended than females (63.5% vs. 50.4%; p = 0.045). No significant differences were observed in literacy (59.8% literate among non-attendees vs. 53.5% among attendees; p = 0.33), indigenous ethnicity (56.9% vs. 56.9%; p = 0.99), or religious affiliation (p = 0.60). The sociodemographic characteristics of the attendees are presented in S2 Table.

**Table 2:** Baseline Characteristics of participants by Attendance Status (Intervention Group, n = 232).

Characteristic	Not Attended (n = 100)	Attended (n = 132)	p-value
**Age, mean (SD)**	60.8 ± 10.2	59.6 ± 10.9	0.25
**Gender, n (%)**			0.045
Male	42 (36.5%)	73 (63.5%)	
Female	58 (49.6%)	59 (50.4%)	
**Education, n (%)**			0.332
Illiterate	47 (40.2%)	70 (59.8%)	
Literate	53 (46.5%)	61 (53.5%)	
**Ethnicity, n (%)**			0.993
Brahmin/Chettri	44 (43.1%)	58 (56.9%)	
Indigenous	56 (43.1%)	74 (56.9%)	
**Religion, n (%)**			0.602
Hindu	70 (45.2%)	85 (54.8%)	
Buddhist	28 (61.6%)	45 (38.4%)	
Christian	2 (50.0%)	2 (50.0%)	

### Adoption and implementation

Adoption was universal, with 100% of health facilities expressing willingness to deliver the FCHV-led hypertension management (Table 3). For implementation fidelity, 76.5% (101/132) of attendees received the full intended dose by attending at least four sessions over three months. Session attendance was distributed as follows: 42.4% attended all six sessions, 12.1% attended five sessions, 21.9% attended four sessions, 3.8% attended three sessions, 16.7% attended two sessions, and 3.0% attended only one session. For FCHV-led hypertension group meetings, 71.1% (145/204) achieved high participation, defined as ≥70% of group members being present.

**Table 3:** Facility-level adoption and implementation fidelity of the FCHV-led hypertension intervention over the 3-month implementation period.

Measure/ Definition	Frequency (%) n = 132
**Facility-level adoption**	100% (all 6 health facilities)
**Implementation Fidelity**	
Number of intended group attendance, mean ± SD	4.50 ± 1.59
Participants attending ≥4 sessions over 3 months	101 (76.5)
Attended all 6 sessions	42.4%
Attended 5 sessions	12.1%
Attended 4 sessions	21.9%
Attended 3 sessions	3.8%
Attended 2 sessions	16.7%
Attended 1 session	3.0%
Groups with ≥70% member attendance	145 (71.1)

There was no significant difference in systolic blood pressure between the intervention and control groups (primary analysis p = 0.85; sensitivity analysis p = 0.68) (Table 4). The intervention significantly improved hypertension-specific knowledge (HKL score) in both the primary analysis (adjusted mean difference 2.2 points, 95% CI 0.6–3.7, p < 0.01) and the sensitivity analysis (2.3 points, 95% CI 0.8–3.8, p = 0.02). Overall adherence, measured by the Hill-Bone total score, also improved significantly (primary analysis: −3.0 points, 95% CI −5.2 to −0.8, p < 0.01; sensitivity analysis: −3.2 points, 95% CI −4.5 to −1.9, p < 0.001).

**Table 4:** Baseline and endline outcomes in intervention and control groups with effect estimates from primary and sensitivity analyses.

Outcome	Baseline mean±SD/ n(%) intervention n = 232	Basline mean±SD/ n(%) Control n = 288	Endline (mean±SD n(%) intervention n = 203	Endline (mean±SD n(%) Control n = 250	Primary analysis Mean difference /RR	P-value	Sensitivity analysis Mean difference/RR	P-value
**SBP (mm Hg)**	135.7 ± 18.3	134.2 ± 14.3	135.3 ± 17.0	133.3 ± 15.3	0.3 (-3.6, 4.4)	0.85	0.7(-2.5,3.9)	0.68
**DBP (mm Hg)**	87.6 ± 10.9	85.8 ± 10.3	86.9 ± 11.5	83.7 ± 10.3	1.5 (-0.8, 3.8)	0.19	1.7(-0.2,3.5)	0.08
GDR score*	12.0 ± 1.1	11.9 ± 1.1	8.1 ± 1.5	8.1 ± 1.4	0.0 (-0.4, 0.4)	0.99	0.1(-0.3,0.4)	0.84
HKL score*	13.9 ± 4.3	13.8 ± 5.4	15.3 ± 4.3	13.8 ± 4.9	2.2 (0.6, 3.7)	<0.01	2.3(0.8,3.8)	0.02
Hill-Bone score**	19.9 ± 4.4	19.0 ± 3.5	19.4 ± 3.7	22.5 ± 5.9	-3.0 (-5.2, -0.8)	<0.01	-3.2(-4.5,-1.9)	<0.001
Medication adherence score **	11.3 ± 3.5	10.6 ± 2.3	10.8 ± 3.1	12.7 ± 4.8	-1.7(-3.7, 0.4)	0.11	1.8(-3.1,0.5)	0.01
**BMI**								
Normal	96 (41.6)	79 (38.9)	132 (45.9)	119 (47.6)	Ref		Ref	
Overweight	135 (58.4)	124 (61.1)	155 (54.0)	131 (52.4)	1.1(0.93,1.2)	0.34	1.1(0.94,1.2)	0.29
**Hypertension**								
Uncontrolled	96 (57.3)	79 (47.9)	132 (51.7)	119 (44.4)	Ref			
Controlled	135 (42.5)	124 (52.1)	155 (48.3)	131 (55.6)	0.9 (0.8, 1.1)	0.39	0.9 (0.8,1.0)	0.16

*(higher score represents better knowledge) **(lower score represents better compliance); Sensitivity analyses excluding outliers in outcome of change.

GDR: Global Dietry Recommendation; HKL score: Hypertension Knowledge score; BMI: Body mass index; RR:Relative Risk; SD: Standard deviation.

No significant intervention effects were observed for diastolic blood pressure (p = 0.19), dietary score (GDR score; p = 0.99), BMI category (p = 0.34), or hypertension control status (p = 0.39) in either the primary or sensitivity analyses. The medication adherence subscale was also not significant in the primary analysis (p = 0.11) but became significant after excluding outliers in the sensitivity analysis (adjusted mean difference −1.8 points, 95% CI −3.1 to −0.5, p = 0.01).

## Discussion

The study aimed to assess the effectiveness and implementation outcomes of an FCHV-led hypertension control intervention in rural Nepal and its effects on systolic blood pressure. The intervention achieved moderate reach and non-attendance was attributed to several contextual constraints: infrastructure disruptions (roadways, electricity, and telecommunications) caused by torrential rainfall, and the seasonal or long-term migration of working-age adults—a demographic trend well-documented in rural Nepal [59]. Importantly, attendees and non-attendees were similar in most sociodemographic characteristics (age, education, ethnicity, religion), but a significant difference was observed by gender. Given that FCHVs in Nepal are an exclusively female cadre with a programmatic focus historically centered on women and children (maternal, newborn, and child health; family planning; women’s empowerment), higher male participation is a particularly noteworthy achievement. This finding demonstrates that the intervention successfully engaged men in a health program traditionally perceived as female-oriented, reflecting broad appeal across gender groups and contributing to greater gender inclusivity in community health outreach. Furthermore, the group-based format may have further minimized differential participation by offering predictable, collective meeting opportunities, helping ensure more equitable engagement across subgroups.

Among attendees, those who participated in four or more sessions showed demographic profiles similar to those who attended less than four sessions. This similarity may reflect barriers to sustained participation that were primarily structural—such as inflexible scheduling, childcare responsibilities, transportation constraints, and competing work or family demands—rather than individual-level factors such as motivation or attitudes.

Despite the limited literacy of FCHVs, they were able to deliver content, aligning with evidence on the practicality and inclusiveness of task-shifting strategies in similar LMIC settings [41,59]. The use of a standard flipchart in every bi-monthly session may have contributed to consistency and quality by offering structured, easy-to-follow visual cues that reduced variation in message delivery [44]. Logistical support including functional BP machines and simplified educational materials, and coordination between FCHVs and health workers may have contributed to enhancing FCHV’s confidence, credibility, and session uniformity [21].

Adoption was exceptionally high, with all participating health facilities willing to implement the intervention. This strong organizational support was likely influenced by prior NCD-related training among health workers and endorsements from municipal authorities [26].

The intervention did not result in statistically significant reductions in systolic or diastolic blood pressure in the primary analysis. Modest mean reductions were observed in the intervention arm, particularly among participants with uncontrolled hypertension. However, these reductions were not statistically significant, likely due to limited statistical power from small subgroup sample sizes.One important explanation for the modest overall effect is that our study’s inclusion criteria required baseline BP ≥ 130/80 mmHg, resulting in a mean baseline SBP of approximately 135 mmHg. According to the “law of initial value” [60,61], greater absolute reductions are expected when starting from higher baseline levels [62,63]. In contrast, several LMIC community health worker-led studies that reported significant BP reductions used a higher inclusion threshold of ≥140/90 mmHg, leading to substantially higher baseline values [42,64,65]. Consistent with this principle, a post-hoc stratified analysis (S3 Table) within our own data showed that participants with higher baseline BP (≥140 mmHg) achieved reductions, whereas those with lower baseline BP showed no reduction. These opposing effects explain the null overall finding and support the interpretation that the intervention is effective in populations with higher baseline BP (≥140 mmHg), similar to the inclusion criteria used in other trials.

Second, the relatively short three-month duration of our intervention may have been insufficient to achieve sustained physiological changes, particularly when compared to similar interventions in Nepal with longer implementation periods—such as the COBIN trial [18] and urban multicomponent CHW-led programs [66]—which modest BP reductions. This aligns with broader evidence that meaningful behavioral and clinical improvements often require higher intervention “dose” and sustained, longer-term engagement [13,67].

Third, immediately after the training of FCHVs) and the initiation of intervention activities by some FCHV-led groups, massive floods and landslides on October 2024, disrupted transportation networks, displaced residents and deaths of people in the catchment area, and severely impeded access, participation, and program delivery [59]. Subsequently, the intervention period coincided with major cultural festivals (Dashain and Tihar) of the dominant ethnic group in the area. During this time, most planned group-based sessions were delayed, as community priorities shifted toward festival-related activities and travel. As a result, opportunities for sustained group engagement, peer interaction, and reinforcement of key behavior change messages were limited. These contextual disruptions likely contributed to the limited behavioral and clinical improvements observed.

Although the intervention did not produce significant changes in clinical outcomes (such as blood pressure), the primary analysis demonstrated clear improvements in hypertension-related knowledge and Hill-Bone adherence scores. These findings are consistent with a recent study from urban Nepal, which showed that CHW-led health education programs effectively increase awareness and enhance treatment compliance among participants [66]. Knowledge improvement, however, did not translate to show significant improvement in diet, physical activity, or BMI—consistent with literature indicating that knowledge alone is rarely sufficient to drive behavioral change [68]. In addition, the short duration of the intervention and disruption in intervention delivery may have further limited the translation of improved knowledge into measurable behavioral and anthropometric outcomes.

Evidence from similar interventions shows that behavior change is strengthened by interactive learning, peer support, and relatable storytelling, as illustrated by CHW experiences in LMIC settings [69–71]. However, external barriers in Namobuddha Municipality—a rural, hilly area in reliant on subsistence and agriculture—likely restricted participants’ ability to apply new knowledge on hypertension management. These include limited year-round access to diverse, nutrient-rich healthy foods (e.g., fruits, vegetables etc.) due to seasonal availability, and economic pressures despite its farming hub status; unsupportive home environments may have shaped by traditional high-salt/carbohydrate diets, and competing familial demands [72].

The reliance on FCHVs in Nepal remains a notable programmatic strength, aligning with growing evidence of their pivotal role in strengthening primary care systems, particularly in rural and hard-to-reach areas [23,71]. Consistent with studies from Nepal and other low- and middle-income countries (LMICs), CHW-led models have demonstrated strong acceptability and effectiveness in promoting lifestyle changes and supporting non-communicable disease (NCD) management [13,42,65]. Despite the culturally tailored, competency-based training and the provision of blood pressure monitors addressing key structural and capacity-related barriers faced by FCHVs and enabling them to deliver blood pressure monitoring, behavior change counseling, and follow-up support, the overall intensity and duration of the intervention were likely insufficient to produce meaningful behavioral and clinical changes.

Implementation was further challenged by contextual disruptions, including natural disasters and major cultural festivals, which reduced the frequency and continuity of group sessions and limited participants’ exposure to core behavior change components [22]. In addition, implementation strategies were deployed without a structured supervisory mechanism, potentially contributing to low implementation fidelity, particularly in the organization of group counseling sessions, documentation and reporting, and audit-and-feedback processes of intervention activities between FCHVs and facility-level health workers.

While the adopted strategies improved FCHVs’ confidence, strengthened community engagement, and facilitated knowledge translation, the modest effects suggest that FCHV-led approaches require further strengthening. As indicated by stakeholder consultations and ongoing formal and informal interactions, enhancements such as intensified supervision, stronger integration with facility-based care, and targeted strategies to address external and contextual barriers may be necessary to achieve meaningful blood pressure reductions and to support scalability in similar resource-constrained settings.

The three-month intervention was too short to assess long-term behavioral and clinical outcomes. Although some improvements in treatment adherence were observed, these findings should be interpreted cautiously, as adherence was self-reported, which may have led participants to overstate adherence to meet perceived expectations [66–68].

## Conclusion

This study demonstrates that a FCHV-led hypertension prevention intervention, supported by competency-based training, provision of monitoring tools and IEC materials, and a structured learning collaborative, was feasible and implemented in a primary healthcare in rural Nepalese context. the intervention achieved moderate reach while adoption was universal across participating health facilities. Implementation fidelity was reasonable, with FCHVs delivering counselling and BP monitoring as intended despite varying literacy levels. The intervention led to improvements in hypertension-related knowledge and self-reported adherence, key intermediate outcomes for blood pressure control. However, these gains did not result in significant reductions in blood pressure, dietary practices, BMI and hypertension control over three months. Overall, the intervention strengthened community engagement, enhanced FCHV capability, and demonstrated the viability of CHW-led hypertension prevention and control efforts in resource-constrained settings.

Future FCHV-led hypertension interventions in rural Nepal should incorporate a qualitative component to gain deeper insights into participants’ experiences, barriers to behavior change, and facilitators of group engagement. Studies should extend intervention duration to 6–12 months with periodic booster sessions and longer follow-up to assess maintenance of effects. Future studies should also evaluate the role of ongoing supervision, stronger integration with health facilities, and additional support for medication access and home blood pressure monitoring. Research should further explore strategies to address common implementation barriers, including flexible scheduling, promotion of local, and enhanced peer-interactive elements. Furthermore, future studies should assess the effectiveness of contingency strategies such as phone-based follow-ups and remote support during disruptions to in-person activities. Finally, since this study single municipality based study, generalizability remains low, so future research should involve multiple sites to ensure broader applicability.

## Supporting information

S1 Fig Implementation Research Logic Model of the study. (TIF)

S1 Table As-treated analysis adjusting for baseline values for study outcomes. (DOCX)

S2 Table Sociodemographic characteristics of participants according to intervention dose (low versus high session attendance). (DOCX)

S3 Table Mean change in systolic blood pressure according to baseline systolic blood pressure category among intervention group participants. (DOCX)

S1 Checklist CONSORT 2025 checklist. Reproduced under the Creative Commons Attribution 4.0 International License.
(DOCX)

S1 Data De-identified participant-level dataset used for analysis. (XLSX)
