# Supplementary figures and images for: Female Community Health Volunteer-led intervention for hypertension prevention and control in rural Nepal: A hybrid type 2 effectiveness-implementation design
Source: PLOS Glob Public Health. 2026 Jul 6;6(7):e0006057. doi: 10.1371/journal.pgph.0006057 (PMC13336210; doi:10.1371/journal.pgph.0006057)

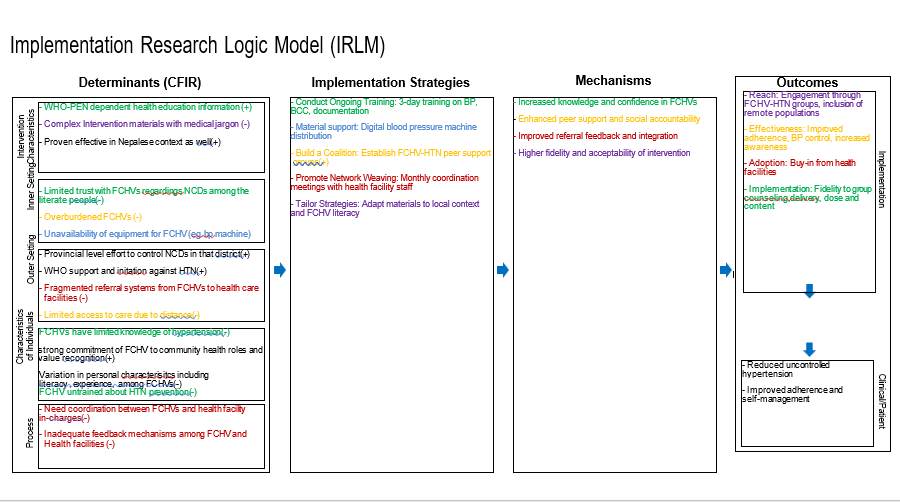

Supplement: S1 Fig — (TIF) [file pgph.0006057.s001.tif]
