# Supplementary material for: Female Community Health Volunteer-led intervention for hypertension prevention and control in rural Nepal: A hybrid type 2 effectiveness-implementation design
Source: PLOS Glob Public Health. 2026 Jul 6;6(7):e0006057. doi: 10.1371/journal.pgph.0006057 (PMC13336210; doi:10.1371/journal.pgph.0006057)
Supplement: S1 Table — (DOCX) [file pgph.0006057.s002.docx]

**S1 Table: As-Treated analysis adjusting the baseline values**

| **Outcome** | **Estimate (95% CI)** | **P-value** |
| --- | --- | --- |
| **Global Dietry Recommendation Score** |  |  |
| No sessions (Ref) | Ref | – |
| Low sessions | 0.16 (-0.68, 0.99) | 0.714 |
| High sessions | 0.38 (0, 0.75) | 0.049 |
| **Hypertension Knowledge Score** |  |  |
| No sessions (Ref) | Ref | – |
| Low sessions | 1.25 (-1.03, 3.53) | 0.283 |
| High sessions | 1.04 (-0.29, 2.36) | 0.124 |
| **Hill-Bone Total Adherence Score** |  |  |
| No sessions (Ref) | Ref | – |
| Low sessions | -0.43 (-3.04, 2.17) | 0.744 |
| High sessions | 0.15 (-1.17, 1.47) | 0.828 |
| **Medication Adherence Subscale** |  |  |
| No sessions (Ref) | Ref | – |
| Low sessions | 0.19 (-2.13, 2.50) | 0.876 |
| High sessions | 0.45 (-0.40, 1.29) | 0.300 |
| **Systolic Blood Pressure (mm Hg)** |  |  |
| No sessions (Ref) | Ref | – |
| Low sessions | 2.54 (-0.24, 5.33) | 0.074 |
| High sessions | -0.31 (-2.38, 1.77) | 0.772 |
| **Diastolic Blood Pressure (mm Hg)** |  |  |
| No sessions (Ref) | Ref | – |
| Low sessions | 2.21 (-0.36, 4.77) | 0.092 |
| High sessions | -0.73 (-2.03, 0.56) | 0.268 |
| **BMI (Overweight/Obese vs Normal)** |  |  |
| No sessions (Ref) | Ref | – |
| Low sessions | RR 1.10 (0.98, 1.25) | 0.105 |
| High sessions | RR 1.11 (0.97, 1.27) | 0.124 |
| **Control Status (Controlled vs Not)** |  |  |
| No sessions (Ref) | Ref | – |
| Low sessions | RR 0.95 (0.68, 1.33) | 0.750 |
| High sessions | RR 1.02 (0.91, 1.13) | 0.765 |
