# Supplementary material for: Female Community Health Volunteer-led intervention for hypertension prevention and control in rural Nepal: A hybrid type 2 effectiveness-implementation design
Source: PLOS Glob Public Health. 2026 Jul 6;6(7):e0006057. doi: 10.1371/journal.pgph.0006057 (PMC13336210; doi:10.1371/journal.pgph.0006057)
Supplement: S2 Table — (DOCX) [file pgph.0006057.s003.docx]

**S2 Table: Sociodemographic characteristics of those who attended the sessions.**

| **Characteristic** | **Low Dose (n=31)** | **High Dose (n=101)** | **Total** | **p-value** |
| --- | --- | --- | --- | --- |
| Gender, n (%) |  |  |  | 0.637 |
| Male | 16 (51.6%) | 57 (56.4%) | 73 (55.3%) |  |
| Female | 15 (48.4%) | 44 (43.6%) | 59 (44.7%) |  |
| Education, n (%) |  |  |  | 0.668 |
| Illiterate | 15 (50.0%) | 55 (54.5%) | 70 (53.4%) |  |
| Literate | 15 (50.0%) | 46 (45.5%) | 61 (46.6%) |  |
| Ethnicity, n (%) |  |  |  | 0.278 |
| Brahmin/Chhettri | 11 (35.5%) | 47 (46.5%) | 58 (43.9%) |  |
| Disadvantaged/Indigenous | 20 (64.5%) | 54 (53.5%) | 74 (56.1%) |  |
| Religion, n (%) |  |  |  | 0.393 |
| Hindu | 22 (71.0%) | 63 (62.4%) | 85 (64.4%) |  |
| Buddhist | 8 (25.8%) | 37 (36.6%) | 45 (34.1%) |  |
| Christian | 1 (3.2%) | 1 (1.0%) | 2 (1.5%) |  |
