# Supplementary material for: Female Community Health Volunteer-led intervention for hypertension prevention and control in rural Nepal: A hybrid type 2 effectiveness-implementation design
Source: PLOS Glob Public Health. 2026 Jul 6;6(7):e0006057. doi: 10.1371/journal.pgph.0006057 (PMC13336210; doi:10.1371/journal.pgph.0006057)
Supplement: S3 Table — (DOCX) [file pgph.0006057.s004.docx]

**S3 Table : Mean change in systolic blood pressure according to baseline systolic blood pressure category among participants in the intervention group**

| **Baseline Systolic Blood Pressure Category** | **Intervention Group (n)** | **Mean Change in SBP, Estimate (95% CI)** |
| --- | --- | --- |
| <140 mmHg | 131 | 3.91 (1.69 to 6.12) |
| ≥140 mmHg | 72 | -8.54 (-12.92 to -4.16) |
