# Supplementary material for: Female Community Health Volunteer-led intervention for hypertension prevention and control in rural Nepal: A hybrid type 2 effectiveness-implementation design
Source: PLOS Glob Public Health. 2026 Jul 6;6(7):e0006057. doi: 10.1371/journal.pgph.0006057 (PMC13336210; doi:10.1371/journal.pgph.0006057)
Supplement: Attachment — Submitted filename: Response to Reviewers.docx [file pgph.0006057.s008.docx]

**Response to Reviewers**

Dear Editor and Reviewers,

We sincerely thank the reviewers for their constructive and insightful comments on our manuscript. These comments have been extremely helpful in improving the clarity, rigor, and overall quality of our work. We have carefully revised the manuscript in response to all suggestions.

**Summary of Major Revisions**

In response to the reviewers’ comments, the following key revisions were made:

- Clarified the discrepancy between our findings and previous studies on FCHV-led hypertension interventions, including contextual, methodological, and follow-up duration differences.
- Strengthened justification of study setting and explicitly acknowledged limitations in generalizability due to single-site design.
- Clarified cluster randomization procedures and allocation of FCHVs at facility level.
- Corrected errors and inconsistencies, including removal of duplicated values and harmonization of outcome definitions.
- Revised statistical reporting by adding p-values for baseline comparisons and clarifying analytical approach using GEE with clustering adjustment.
- Added detailed explanation for missing data and reasons for loss to follow-up.
- Clarified differences between eligibility criteria (≥130/80 mmHg) and outcome definition (<140/90 mmHg).
- Expanded discussion on baseline BP influence using the “law of initial value” and added post hoc stratified analysis interpretation.
- Improved language, terminology consistency, and reduced first-person pronouns while maintaining clarity in methodological descriptions.
- Revised Discussion section to reduce repetition of results and improve interpretive depth.

**Details of Response**

**Reviewer #1**

*Comment 1:*

**The literature has shown that FCHV-led approach can improve hypertension outcomes. However, current study concluded with different conclusions. Please discuss their discrepancies.**

Response:

Thank you for your comment. You are right that this is an important observation. Previous studies have demonstrated significant blood pressure reduction with FCHV-led interventions. However, our study did not observe a statistically significant change in blood pressure despite improvements in knowledge and medication adherence. This difference may be explained by several factors: (1) the relatively short follow-up period (3 months), which may have been insufficient to observe measurable changes in blood pressure; (2) baseline blood pressure levels being relatively close to the controlled range, thereby limiting the magnitude of detectable reduction; and (3) contextual differences in population characteristics and implementation settings. In addition, the study population, setting, and duration of follow-up in our study are not directly comparable with most previous studies. We have incorporated these explanations into the Discussion section with supporting references .

**Comment 2:**

**Line 128. The site selection is based on their readiness for community-based intervention. Please comment on its representativeness.**

Response:

Thank you for this insightful comment. The study site was purposively selected based on readiness of municipality authority to implement community-based interventions. Regarding the readiness, the selected municipality shares key demographic, geographic, and health system characteristics with many rural settings in Nepal, supporting the generalization of findings to similar contexts, however this single site study has low power for generazability in large scale, so multi-site has been suggested in conclusion section (i.e line number 557 – 561) and acknowledged as a limitation.

Include in manuscript as *: Furthermore, future studies should assess the effectiveness of contingency strategies such as phone-based follow-ups and remote support during disruptions to in-person activities. Finally, since this study single municipality-based study, generalizability remains low, so future research should involve multiple sites to ensure broader applicability*.

**Comment 3:**

**Line 142. Please clarify how the 34 FCHVs are distributed among 6 facilities. Are they randomly distributed?**

Response:

Thank you for your comment. In this cluster randomized trial, the unit of randomization was the health facility. The 34 Female Community Health Volunteers (FCHVs) were not randomly allocated; rather, they were pre-assigned to their respective health facilities based on existing government-defined catchment areas. The research team had no role in assigning FCHVs to intervention or control arms. An independent statistician generated the random allocation sequence and assigned the six health facilities (clusters) to either the intervention or control arm.

Revised statement on revised manuscript is as follow (line number 141-143):

“*We included FCHVs who were pre-assigned to their respective health facilities based on existing government-defined catchment areas of Namobuddha Municipality. We enrolled the 34 FCHVs working under the 6 different health facilities of the intervention arm, and who were willing to participate in this study*.

**Comment 4:**

**Line 317. Drop repeated 13.2%**

Response:

Thank you for identifying this duplication. The repeated value has been removed.

**Comment 5:**

**Intervention and control groups were from different facilities, which may lead to confounding or biased results.**

Response:

We agree with this concern. We used a cluster randomized design, with health facilities as the unit of randomization, to minimize contamination between participants. However, allocation at the cluster level may introduce confounding due to baseline differences between facilities. To address this, we compared baseline characteristics across study arms and conducted intention-to-treat analyses using generalized estimating equations (GEE), accounting for clustering at the facility level and adjusting for baseline outcome values and relevant covariates. These details have been well mentioned in line number 293 to 294.

**Comment 6:**

**Table 1. It would be informative to provide the p-values for the comparison of the sample characteristics between intervention and control groups.**

Response:

We have revised Table 1 to include p-values for comparisons between intervention and control groups.

**Comment 7:**

**The primary effectiveness outcome was the change in mean systolic blood pressure at 3 months (line 240), which is different from “endline systolic blood pressure” (line 288) in statistical analysis. This needs to be clarified.**

Response:

Thank you for pointing out this inconsistency. We have revised the manuscript to ensure consistent terminology. The primary outcome is now clearly defined as the change in mean systolic blood pressure from baseline to 3 months, and all related sections have been aligned accordingly.

**Comment 8:**

**Need to provide the reason of missingness.**

Response:

We have added details regarding missing data in the Methods and Results sections. The primary reasons for missingness included loss to follow-up, temporary migration, and unavailability during endline assessment.

Revised statement in revised manuscript is as follow:

“*During follow-up, 12.5% (n = 29) of participants in the intervention arm were lost to follow-up, primarily due to withdrawal of consent (n = 10), migration (n = 9), and loss of contact (n = 10).In the control arm, 13.2% (n = 38) of participants were lost, due to withdrawal of consent (n = 12), migration (n = 7), and loss of contact (n = 20).”*

**Comment 9:**

**Hypertension control definition discrepancy (SBP <140/90 vs inclusion ≥130/80). Please clarify.**

Response:

We appreciate this important point. Thank you for this observation. The difference reflects the distinction between who we included and what we measured. We included participants with blood pressure ≥130/80 mmHg to capture both prehypertensive and hypertensive individuals, as the study focused on both prevention and control of hypertension.

However, hypertension control (<140/90 mmHg) was used as an outcome based on standard clinical practice and was assessed among participants with hypertension.

**Comment 10:**

**Discussion mentioned that BP reduction is more substantial among individuals with higher baseline. Is this also true in the current study?**

Response:

Thank you for this important observation. Yes, our findings are consistent with this pattern. Our lower inclusion threshold (≥130/80 mmHg) resulted in a modest baseline SBP (~135 mmHg), compared to other LMIC studies (≥140/90 mmHg; baseline ≥140–159 mmHg). In line with the *law of initial value*, this likely explains the smaller overall BP reduction.

A post hoc stratified analysis showed a trend toward greater reduction among those with higher baseline BP, though not statistically significant, likely due to limited sample size and short follow-up.

Ther revised statement in revised manuscript is as follow: *The intervention did not result in statistically significant reductions in systolic or diastolic blood pressure in the primary analysis. Modest mean reductions were observed in the intervention arm, particularly among participants with uncontrolled hypertension. However, these reductions were not statistically significant, likely due to limited statistical power from small subgroup sample sizes.One important explanation for the modest overall effect is that our study’s inclusion criteria required baseline BP ≥130/80 mmHg, resulting in a mean baseline SBP of approximately 135 mmHg. According to the “law of initial value” (60,61), greater absolute reductions are expected when starting from higher baseline levels (62,63). In contrast, several LMIC community health worker‑led studies that reported significant BP reductions used a higher inclusion threshold of ≥140/90 mmHg, leading to substantially higher baseline values (42,64,65). Consistent with this principle, a post‑hoc stratified analysis (Supplementary) within our own data showed that participants with higher baseline BP (≥140 mmHg) achieved reductions, whereas those with lower baseline BP showed no reduction. These opposing effects explain the null overall finding and support the interpretation that the intervention is effective in populations with higher baseline BP (≥140 mmHg), similar to the inclusion criteria used in other trials*

**Reviewer #2**

**Comment:**

**Prefer to use demonstrative pronouns like "the study" instead of "we" in the methodology section.**

Response:

Thank you for this valuable suggestion. We acknowledge the reviewer’s preference for minimizing first-person pronouns in the methodology section. However, we intentionally retained active voice in several sections because it improves clarity, readability, and transparency in describing study procedures and analytical decisions. Contemporary scientific writing guidelines increasingly support the use of active voice where appropriate, particularly in reporting methodological processes. However, we revised selected sentences to reduce repetitive use of “we” and incorporated more neutral constructions as suggested “the study” where these improved readability without compromising clarity.

**Reviewer #3**

**Comment 1:**

**The discussion section can be elaborated with further relevant references.**

Response:

Thank you for this valuable suggestion. We have carefully reviewed the Discussion section and ensured that relevant and context-specific references, particularly those related to group counselling through community health workers and recent evidence from Nepal, are included. Given the limited availability of contextually comparable studies, we prioritized the most pertinent literature. We believe that adding additional references beyond these would provide limited incremental value and may reduce the focus and clarity of the discussion.

**Comment 2:**

**Summarization of the results should be avoided in the discussion section.**

Response:

Thank you for your comment. We have revised the text in the Discussion section to avoid repetition of results, and moved beyond the raw results to the interpretation, comparison with existing literature, and implications.

**Comment 3:**

**Limitations and delimitations of the study could be separated from the discussion section.**

Response:

Thank you for this suggestion. As per the CONSORT Statement (Item 30- <https://www.consort-spirit.org/> ), limitations are recommended to be explicitly addressed within the Discussion rather than as a separate section. Following this standard reporting practice, we have retained the limitations within the Discussion while improving their clarity and structure.

We sincerely thank the reviewers for their valuable feedback, which has significantly strengthened the manuscript.

**Bihungum Bista**

Corresponding Author
